# The European Classical Swine Fever Virus Database: Blueprint for a Pathogen-Specific Sequence Database with Integrated Sequence Analysis Tools

**DOI:** 10.3390/v8110302

**Published:** 2016-11-07

**Authors:** Alexander Postel, Stefanie Schmeiser, Bernd Zimmermann, Paul Becher

**Affiliations:** 1 EU and OIE Reference Laboratory for Classical Swine Fever, Institute of Virology, Department of Infectious Diseases, University of Veterinary Medicine, 30559 Hannover, Germany; Alexander.Postel@tiho-hannover.de (A.P.); st.schmeiser@gmail.com (S.S.); 2 Bernd Zimmermann Software Development, IT Consulting and Support, 31542 Hannover, Germany; bernd@bernd--zimmermann.de

**Keywords:** virus, sequence, database, disease control, classical swine fever

## Abstract

Molecular epidemiology has become an indispensable tool in the diagnosis of diseases and in tracing the infection routes of pathogens. Due to advances in conventional sequencing and the development of high throughput technologies, the field of sequence determination is in the process of being revolutionized. Platforms for sharing sequence information and providing standardized tools for phylogenetic analyses are becoming increasingly important. The database (DB) of the European Union (EU) and World Organisation for Animal Health (OIE) Reference Laboratory for classical swine fever offers one of the world’s largest semi-public virus-specific sequence collections combined with a module for phylogenetic analysis. The classical swine fever (CSF) DB (CSF-DB) became a valuable tool for supporting diagnosis and epidemiological investigations of this highly contagious disease in pigs with high socio-economic impacts worldwide. The DB has been re-designed and now allows for the storage and analysis of traditionally used, well established genomic regions and of larger genomic regions including complete viral genomes. We present an application example for the analysis of highly similar viral sequences obtained in an endemic disease situation and introduce the new geographic “CSF Maps” tool. The concept of this standardized and easy-to-use DB with an integrated genetic typing module is suited to serve as a blueprint for similar platforms for other human or animal viruses.

## 1. Introduction

The worldwide exchange of genome data from viruses causing severe transboundary diseases is urgently needed in human and veterinary medicine to perform molecular epidemiological investigations in the case of epidemics. Advances in classical molecular techniques such as improved complementary DNA (cDNA) synthesis and PCR performance have increased the length and quality of sequence reads achieved by Sanger sequencing [1,2,3]. Moreover, the implementation of next-generation sequencing (NGS) techniques has revolutionized the genetic typing of pathogens in terms of sensitivity and speed and allows for the rapid determination of complete genome sequences of many viruses and bacteria [4,5]. The World Organisation for Animal Health (OIE) has recently recommended the inclusion and validation of NGS into the Terrestrial Manual and requests a platform for the collection and management of viral genomic sequences, including genotype assignment with a connection to existing virus-specific sequence databases [6].

Classical swine fever (CSF) is one of the most important pig diseases worldwide and is, due to its severe socio-economic impacts, notifiable to the European Union (EU) and the OIE. Successful containment of CSF outbreaks relies on rapid diagnosis, identification of infection routes, efficient control measures, and the exchange of information on regional, national, and international levels. The sequence database (DB) of the EU and OIE Reference Laboratory for classical swine fever (EURL CSF) was a pioneer project, already established in 2000 as a web interface platform for the exchange of classical swine fever virus (CSFV) genome data (Version 1.0) and was further improved by the integration of an easy-to-use sequencing tool for molecular epidemiology in 2006 (Version 2.0) [7,8]. In past years, laboratory reports of the EURL CSF to the Directorate General for Health and Food Safety (DG SANTE) of the EU commission and to the affected member states have routinely included phylogenetic trees generated with this DB and have therefore provided useful information about the causative virus isolate to the authorities. Until now, the DB included sequences of short genome fragments, namely, a partial 5′ non-translated region (5′NTR, 150 nt), a partial E2 (190 nt), and a partial NS5B (409 nt) encoding fragment, as proposed in the context of a previous initiative to harmonize CSFV sequencing strategies in 2000 [9]. Since then, the analysis of the complete E2 encoding sequence (1119 nt) was found to be much more suitable in particular for epidemiological research studies and for the differentiation of highly similar virus isolates. This strategy has been already applied at the EURL CSF for recent molecular epidemiological studies on CSFV [10,11,12]. In consequence, the sequence DB and the genetic typing module have been re-programmed to facilitate the storage and the phylogenetic analysis of longer genomic sequences. In this study, the structure and the relevant modifications of the CSF-DB are introduced via application examples. Moreover, the possibility of using this DB as a blueprint for other pathogen-specific databases is discussed.

## 2. Materials and Methods

### 2.1. Database (DB) Design

The CSF-DB runs on a Linux server using a MySQL-Server (Version 5.5.50). The Web frontend for maintaining, searching, and accessing the separately running genetic typing module and the new “CSF Maps” tool is written in PHP (Version 5.4.45). The PHP scripts run on an Apache web server (Version 2.2.22). The CSF-DB is available online via http://viro60.tiho-hannover.de/eg/csf/; access is permitted after a password request at csf.eurl@tiho-hannover.de.

### 2.2. Nucleotide Sequencing

CSFV nucleotide sequences were determined as described previously [11]. E2 encoding sequences of the CSF-DB catalogue numbers CSF0088, CSF0183, CSF0605, CSF0631, CSF0804, CSF0818, CSF0819, CSF0869, CSF0934, and CSF1040 were included in the CSF-DB and deposited in GenBank under Acc. Nos. KX687712–KX687721.

### 2.3. Implementation of the “CSF Maps” Tool

The sequence database was extended to allow geocoding (longitude and latitude data) and the subsequent graphical display of outbreak locations. Each master entry and each sequence can be stored with a location text like a city or region name. Location and country information were geocoded using the Google Maps Geocoding API [13]. The resulting longitude and latitude data were stored in the corresponding dataset (also applicable to additional sequences of a master sequence). To visualize locations of isolated CSFV, the function “CSF Maps” was included comprising search categories such as CSF number, year of isolation, country, and the option to search for follow up outbreaks. The results are displayed on a map generated using Google Maps Java-Script API [14].

### 2.4. Improvements of the Genetic Typing Module

The original typing module was implemented in Borland Delphi 7 running on a 32 bit Windows environment. The software was recompiled using a 64 bit Windows environment with a Delphi compiler (RAD Studio XE5; Embarcadero, Austin, Texas, USA) and actual components for data access (MyDAC, CoreLab software development) and web frontend (intraweb components from Atozed-Software). The module was embedded into the maintaining PHP web frontend on the web server but running on a separate computer to speed up computing time. Additional code optimization of the implemented algorithms (Needleman-Wunsch, Smith-Waterman, Neighbor-Joining) was achieved by loop unrolling, via dead code elimination, via the deletion of unused variables, and by restructuring the code.

## 3. Results

### 3.1. Novel Features and Data Provided by the Classical Swine Fever (CSF) DB (CSF-DB)

The design and structure of the existing DB have been modified and improved according to the actual needs of users. In addition to the established short genome fragments in the 5′NTR (150 nt) and E2 encoding region (190 nt), there is now extended storage possibility for sequence data of full E2 gene (1.1 kb), 5′NTR-E2 (3.3 kb), and the complete CSFV genome (12.3 kb). In case of follow-up outbreaks, very similar sequences can be stored under the same catalogue number as “additional sequences” to a “master sequence.” To provide an improved data overview and documentation for the user, it is now possible to print fact sheets of individual virus isolates.

As of 1 July 2016, the DB contains information on a total amount of 1,075 CSFV isolates stored in the virus collection of the EURL CSF (indicated by catalogue numbers beginning with “CSF”). In addition, the DB contains 712 CSFV sequences with no CSFV isolate available (indicated by catalogue numbers beginning with “XXX”), either from CSFV positive sample material, which did not allow virus cultivation, or from sequence data retrieved from Genbank/EMBL data libraries. The DB already harbors over 1000 sequences of the short E2 fragment and 265 full E2 gene sequences, which were generated and added to promote the use of longer sequence regions in CSFV phylogeny (Table 1).

Since 2007, 77 new virus isolates have been included in the DB, mainly from South-East Europe, the Baltic countries, Germany, Asia (including Nepal, Vietnam, and Thailand), Israel, and Cuba. Sequences of all currently known CSFV genotypes comprising the subgenotypes 1.1–1.4, 2.1–2.3 and few sequences from the ancient and rare genotype 3 are stored in the DB (Table 1). Sequences of the genome regions NS5B and 5′NTR-E2 as well as complete genome sequences are currently rarely used for genetic typing of CSFV and therefore are so far only available in the administrator mode. Accession numbers of partial and complete genome sequences available from GenBank can be found in the CSF DB. The release of the complete genome sequences is in preparation and will be part of the next database update.

### 3.2. Visualization of outbreak locations by the “CSF Maps” tool

**Figure 1:**
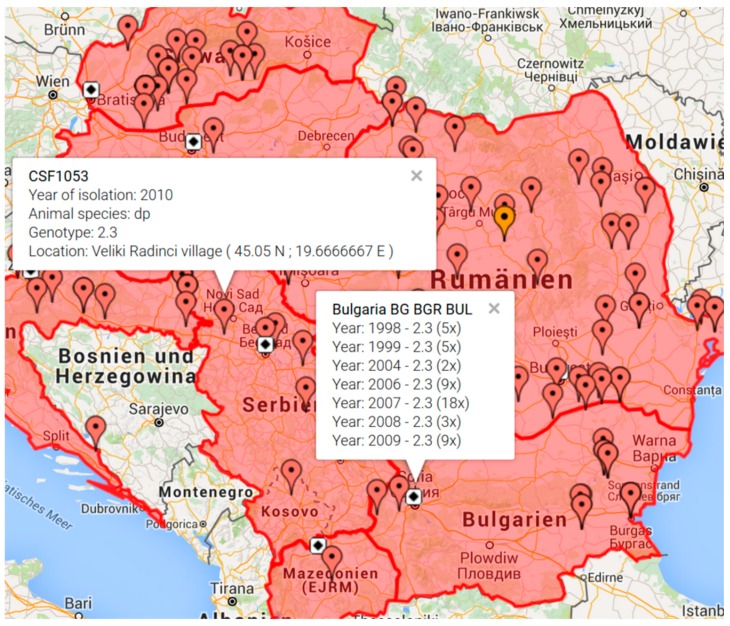
Geographic display of classical swine fever virus (CSFV) isolates available from the Balkan region. Map generated by the “CSF Maps” tool based on Google maps. The map was generated by selecting the countries Albania, Bulgaria, Croatia, Hungary, Macedonia, Montenegro, Romania, Serbia, and Slovakia. The options “show outbreaks”, “no display of isolates with unspecified location”, and “isolation years 1998–2010” were selected. Clicking on the flagged outbreak location provides additional information comprising catalogue number (e.g., CSF1053), year of isolation, animal species (domestic pig (dp) or wildboar (wb)), genotype, and location with longitude and latitude coordinates. Clicking on the capital of a country (symbolized by a white square with black diamond) provides an overview of available isolates from the respective country (e.g., Bulgaria) comprising isolation year, genotype, and number of available database entries.

The new “CSF Maps” tool allows the user to search for all outbreak isolates from a given catalogue number related to a country or year of isolation. It can be chosen whether follow up outbreaks shall be included or not. Each outbreak location is displayed by a colored flag, with each color representing a distinct genotype. By clicking on the colored flag, a popup window provides information about the catalogue number, year of isolation, genotype, and location (Figure 1). Clicking on the capital symbol of a country provides an overview about the number of available database entries ordered by isolation year and CSFV genotype, including a list of isolates with unknown outbreak location (Figure 1).

### 3.3. Novel Features in the Genetic Typing Module

**Figure 2:**
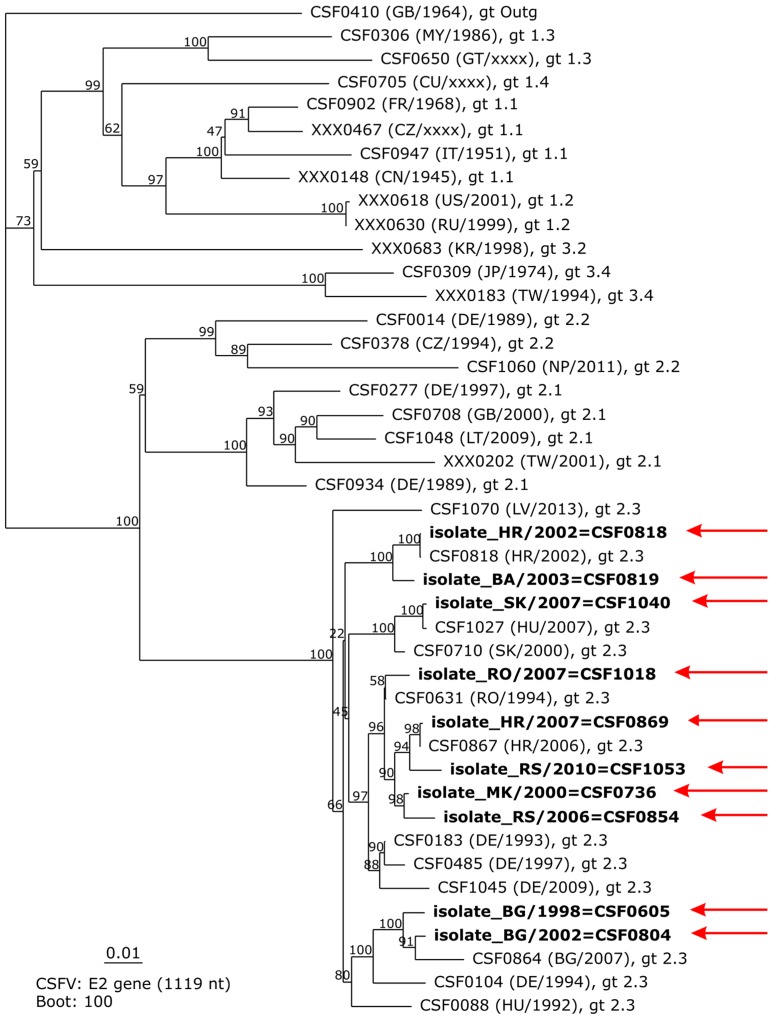
Phylogenetic tree based on E2 encoding sequences (1119 nt) generated by the genetic typing module of the classical swine fever database (CSF-DB). A neighbor-joining tree comprising a pre-defined set of reference sequences and ten additional sequences obtained from endemic CSF disease situations in wild boar in Balkan countries between 1998 and 2010 is shown. Individually added sequences are highlighted in boldface and indicated by red arrows. In the default configuration of the genetic typing module, bootstrap values are calculated for 100 iterations.

The “pairwise alignment” function shows a list of the most similar sequences stored in the DB by giving its catalogue number, country, and nucleotide identity. This tool allows for the identification of sequences that can provide interesting information when added to a subsequent phylogenetic analysis. The genetic typing module allows phylogenetic analysis of up to ten sequences of choice (partial 5′NTR sequences and partial and complete E2 coding sequences), including bootstrap analysis for statistical evaluation. For phylogenetic analysis, newly generated sequences and additional sequences from the CSF-DB can be included, which are integrated in a pre-defined phylogenetic tree of reference sequences and rooted to the sequence of isolate “Congenital Tremor” (CSF0410, GB/1964). The power to discriminate genetically closely related CSFV isolates becomes evident when, say, analyzing highly similar sequences obtained from an endemic disease situation such as that faced in the Balkan region from 1998 to 2010. The phylogenetic tree based on complete E2 coding sequences is supported by high bootstrap values at important nodes defining the different subgenotypes or phylogenetic clades (Figure 2).

To increase the flexibility of epidemiological analyses required within EURL CSF tasks, the reference set of isolates and the parameters for calculation and generation of phylogenetic trees, as well as the output file format (bitmap files for external users), can be modified by the administrator (Newick tree format or scalable vector graphics).

## 4. Discussion

In the majority of laboratories that have access to advanced diagnostic methods such as nucleotide sequencing, the data output in the last decade has significantly increased. However, even in times of international networking for many globally relevant diseases, it is still hardly possible to obtain a structured overview on the geographical distribution of circulating genotypes of pathogens as accentuated and faced by the OIE [6]. By restructuring and enlarging the sequence DB, such a platform is already implemented for CSF and has been improved in capacities, genetic typing possibilities, and flexibility to match the actual needs of public users as well as to support the work and the data management of the EURL CSF.

Established typing of CSFV isolates using short genome fragments, such as the conserved 5′NTR (150 nt) fragment sequences, allows for phylogenetic analysis with restricted resolution and often gives insufficient statistical support when closely related CSFV isolates are analyzed. Phylogenetic analysis based on the genetically variable E2 fragment (190 nt) gives more reliable and timely results. Due to the frequent use of this region for typing in recent decades, applying this strategy is advantageous in that a wealth of sequence information is available. Nevertheless, the increasing availability of longer nucleotide sequences, such as the complete E2 gene (1119 nt) and even full genome sequences (12.3 kB), will further improve the possibilities for high-resolution phylogenetic analyses. The new DB is taking this development into account and—together with the new “CSF Maps” tool—is well suited to illustrate geographic and genetic relationships between isolates, thereby providing an improved tool for disease control. 

By now, the presented database is in its technical functions comparable to other pathogen-specific approaches established for influenza virus, bovine viral diarrhea virus, and foot and mouth disease virus, the latter lacking an integrated tool for phylogenetic analysis [15,16,17]. The Virus Pathogen Resource (ViPR) is a different database project combining information from several primary databases (e.g., the GenBank sequence database). The ViPR covers a broad range of relevant viral human, animal, and zoonotic pathogens (e.g., hepatitis C, Ebola, dengue, and Zika viruses), allowing also for the comparison and phylogenetic analysis of viral sequences, primarily addressing needs for research activities [18,19]. The concept of the presented database is to provide information relevant for a specific pathogen and to focus on the needs with respect to the diagnosis and control of a disease. The possibility of including a certain set of sequences in a predefined phylogenetic tree ensures that the dataset is representative for sequences of different genotypes. Furthermore, the use of defined genomic regions for genetic typing allows for an easy comparison of phylogenetic trees generated by different laboratories. Many statistical methods and programs for sequence and phylogenetic analysis are presently available [20]. The approach presented here is easy to use and ensures consistent quality by the same bio-mathematical procedure and the same reference sequence set among all users. This is of particular importance, as phylogenetic trees are often requested for official diagnostic laboratory reports to national authorities and the EU Commission. The usage of a common DB that provides an integrated sequence analysis tool is helpful for individual laboratories and ensures reliability of sequence analysis results. 

The DB and the integrated genetic typing module are constructed as easy-to-use tools for users worldwide to perform fast and reliable molecular characterization of the pathogen in case of infectious disease outbreaks. Both the DB and the genetic typing module are designed for easy export to other virus systems. The DB has already been successfully applied for bovine viral diarrhea virus and porcine reproductive and respiratory syndrome virus (unpublished), which are also RNA viruses with a relatively small genome size. For DNA viruses with large genomes, e.g., pox or herpesviruses, the calculation of multiple sequence alignments and phylogenetic trees on the basis of complete genome sequences would require high server capacities; this limiting factor is independent of the use of a database. Usually, sequence alignments and phylogenetic trees for such viruses are not calculated for complete genome sequences, but on the basis of partial genomic sequences. Moreover, extending the functionality of the CSF-DB (e.g., no pre-defined reference tree, a changeable number of bootstrap repetitions in phylogenetic analyses, and public access to all stored sequence and isolate information) can easily be done and adapted to the needs and preferences for other applications. Accordingly, the concept of the presented DB can serve as a blueprint for other pathogen-specific databases and sequence analysis platforms.

## Figures and Tables

**Table 1:** Overview on total number of sequence data sets in the classical swine fever (CSF) sequence database.

Genome Region	5′NTR	E2 Fragment	E2 Gene	NS5B	5′NTR-E2	Full Genome
(Nucleotides)	(150)	(190)	(1119)	(409) *	(3.3 k) *	(12.3 k) *
Isolates (CSF No.)	661	557	171	43	46	11
only sequence (XXX No.)	251	525	94	96	44	42
**Total**	912	1082	265	139	90	53

* Available in administrator mode only; NTR: non-translated region.
